# Determining baselines for human-elephant conflict: A matter of time

**DOI:** 10.1371/journal.pone.0178840

**Published:** 2017-06-05

**Authors:** Rocío A. Pozo, Tim Coulson, Graham McCulloch, Amanda L. Stronza, Anna C. Songhurst

**Affiliations:** 1 Department of Zoology, University of Oxford, Oxford, United Kingdom; 2 Ecoexist Project, Maun, Botswana; 3 Applied Biodiversity Science Program, Texas A&M University, College Station, Texas, United States of America; Australian National University, AUSTRALIA

## Abstract

Elephant crop raiding is one of the most relevant forms of human-elephant conflict (HEC) in Africa. Northern Botswana holds the largest population of African elephants in the world, and in the eastern Okavango Panhandle, 16,000 people share and compete for resources with more than 11,000 elephants. Hence, it is not surprising this area represents a HEC ‘hotspot’ in the region. Crop-raiding impacts lead to negative perceptions of elephants by local communities, which can strongly undermine conservation efforts. Therefore, assessing trends in conflict levels is essential to developing successful management strategies. In this context, we investigated the trend in the number of reported raiding incidents as one of the indicators of the level of HEC, and assessed its relationship to trends in human and elephant population size, as well as land-use in the study area. For each of these factors, we considered data spanning historical (since the 1970s) and contemporary (2008–2015) time frames, with the aim of comparing subsequent inferences on the drivers of crop raiding and predictions for the future. We find that the level of reported crop raiding by elephants in the eastern Panhandle appears to have decreased since 2008, which seems to be related to the reduction in agricultural land allocated to people in recent years, more than with human and elephant population size. We show that inferences regarding the drivers of HEC and predictions for the future are dependent on the time span of the data used. Although our study represents a first step in developing a HEC baseline in the eastern Panhandle, it highlights the need for additional multi-scale analyses that consider progress in conservation conflict to better understand and predict drivers of HEC in the region.

## Introduction

Elephant crop raiding (i.e. the consumption of and/or damage to crops) is currently one of the most prevalent forms of conflict between humans and elephants worldwide [1–3]. The on-going expansion of human settlements and activities, in addition to growing elephant populations outside of protected areas in some localities [4–6], has resulted in increased levels of interaction between people and elephants [7]. Crop-raiding in particular has direct impacts on human livelihoods, through the destruction of agricultural crops and nearby properties, as well as injuries to people and in some instances, death [1, 7–13]. Such impacts lead to negative perceptions of elephants by local communities, which in many cases strongly undermine conservation efforts targeted at elephant populations [14–16].

Assessing trends in human-elephant conflict (HEC) impacts [see 17] is essential to identifying key drivers and devising successful management strategies. However, in order to identify changes in HEC patterns, we first need to understand past and present trends. To do this, effective monitoring and evaluation systems are needed in areas of interaction between people and elephants. Monitoring programs provide a means of evaluating progress made towards wildlife conservation [18–19], and one monitoring strategy in elephant conservation is to count and assess crop-raiding incidents reported in affected areas. Nevertheless, the interpretation of observed trends can be dependent on the time frame over which they are measured, which may be constrained by the availability of historical data [20]. A consequence of this is that the reference point against which the current state of HEC impacts is compared, i.e. a baseline, may be arbitrary. Determining a baseline before conservation implementation starts is a key step in the conservation evidence framework [21–22]. Lack of an appropriate “frame of reference” is a widespread issue in the evaluation of conservation actions worldwide [23]. This is particularly relevant to human-wildlife conflict studies [24] for which long-term data on impacts and potential drivers are scarce [25]. In the case of HEC, not only does this affect our understanding of current impacts within a defined region, but it also limits our ability to make robust predictions for future trends.

Botswana holds the largest population of African elephants (*Loxodonta africana*) in the world [4, 6], and the Okavango Delta Panhandle represents a stronghold in terms of increasing elephant numbers and conservation in Africa. Approximately sixteen thousand people currently inhabit the eastern section of the Panhandle [26], sharing and competing for resources with a population of more than eleven thousand elephants [27]. Hence it is not surprising that this area also represents an HEC ‘hotspot’ in the region. Elephant crop-raiding is seasonal, with most incidents occurring throughout the crop-growing and harvesting months [28–29]. Due to the presence of both artificial (e.g. veterinary fencing) and natural barriers (e.g. the permanent Okavango River) to elephant movement, elephant activity is concentrated within this area. Previous studies have suggested that crop-raiding incidents are associated with the spread of human populations, and particular agricultural encroachment into wild areas [2, 8]. It is also believed that an increasing elephant population leads to a higher incidence of crop-raiding events [30–31]. Based on this, it can be expected that the incidence of crop-raiding will increase in the coming years in the eastern Panhandle, yet no study has attempted to verify this assertion by quantifying and predicting trends in human and elephant populations, as well as changes in land-use.

In this study, we first characterise temporal trends in the incidence of reported crop-raiding events by elephants occurring in the eastern Panhandle of Botswana’s Okavango Delta. We then investigate trends in potential drivers of crop-raiding incidence, i.e. human and elephant population sizes, as well as the amount of agricultural land allocated, with the latter trends estimated using data collected over two temporal scales, historical (1970s - 2015) and contemporary (2008–2015). Thirdly, we assess the relationship between each of these drivers and reported crop raiding. Finally, we use the results from the previous steps to predict future levels of crop raiding, comparing the influence of historical and contemporary time scales on predictions.

## Materials and methods

### Study area

**Fig 1:**
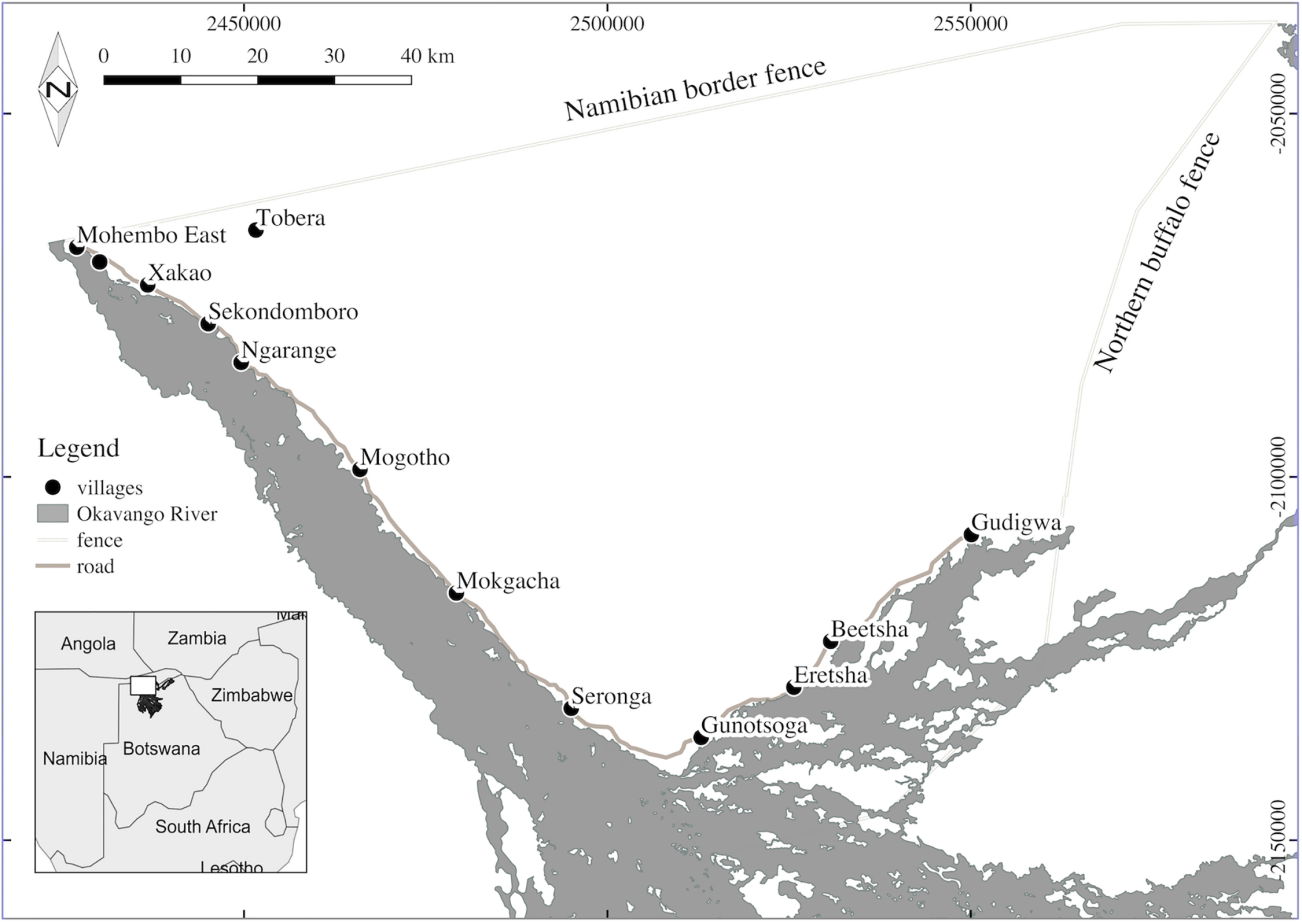
Location of villages in the eastern Okavango Delta Panhandle (Botswana). Circles represent the thirteen villages (i.e. Mohembo East, Kauxwi, Tobera, Xakao, Sekondomboro, Ngarange, Mogotho, Mokgacha, Seronga, Gunotsoga, Eretsha, Beetsha and Gudigwa) along the Okavango River. The small southern Africa inset map shows the location of the study area in northern Botswana in white. This image is not identical to Fig 2 in Songhurst and Coulson (2014), and therefore it is for illustrative purposes only.

The study area is located in the eastern Okavango Delta Panhandle in the Ngamiland District of northern Botswana. The Okavango Delta is formed from the Okavango River that originates in Angola, flows along the Caprivi Strip in Namibia and reaches a tectonic trough in the centre of the Kalahari. The 8,732km^2^ non-protected area in the eastern Okavango is a mixture of agricultural land, people’s settlements and savannah shrubland delimited by the Namibian border to the north, the Okavango River to the southwest and the northern buffalo fence on the south-eastern edge [29] (Fig 1). Deep Kalahari sands cover the majority of the region, with fertile soils near the Okavango River. Vegetation cover is represented mainly by mopane (*Colophospermum mopane*) woodland, *Terminalia sericea* sandveld, mixed marginal floodplain woodland (*Acacia nigrescens* and *Hyphaene petersiana*), acacia woodland (*Acacia erioloba*, *Acacia tortillis*) and perennial swamp woody communities [32]. The Delta has a typical continental climate with annual rainfall of 360–500 mm, mostly concentrated during the wet season (November to April). Daily temperatures vary from 25–35°C during the day to an average of 8°C during the night [33]. The hottest month of the year is October, at the end of the dry season (May to October).

More than 16,000 people live in thirteen villages along the Okavango River from Mohembo East to Gudigwa (Fig 1). The largest village is Seronga with 3,716 inhabitants [26]. Human livelihoods consist mainly of subsistence agriculture. Local farmers cultivate fields in areas from the river edge up to 14km inland. Ploughing takes place at the beginning of the wet season and crops are harvested every year between April and June. The last estimated elephant population size in the study area was 11,760 [27].

### Data collection

In this study, we used data on the size of human and elephant populations, as well as on the area of agricultural land allocated, that had been previously collected in Botswana by the Department of Wildlife and National Parks (DWNP), the Central Statistical Office (CSO, Ministry of Finance and Development Planning), the Land Board Office (Ministry of Agriculture), and the Ecoexist Project. Our study uses data from elephant aerial surveys collected in the eastern Panhandle. These were performed either by the DWNP or by members of the Ecoexist Project. In both cases, the DWNP authorised the collection and use of the aerial survey data [for more details see 27, 29]. This project was under research permit EWT 8/36/4 XXVI (86) issued by the DWNP in May 2014, which allowed use and collection of data pertaining to the study area.

We used estimated elephant population sizes reported by the DWNP between 1991 and 2013. These were combined with independent aerial survey data collected across the study area by the Ecoexist Project [34] (Table 1). In all aerial surveys (i.e. Governmental and independent) population size estimates were obtained using the Jolly Method II [35] for sampling blocks of unequal size and only dry season data was used.

**Table 1:** Human and elephant population size, agricultural land allocated (ALA) and number of reported raid incidents (RRI) from raw and predicted data.

Year	Elephant population			Human population		ALA (ha)			# RRI	
	Raw data	Predicted data	Source	Raw data	Predicted data	Raw data	Predicted data (historical)	Predicted data (contemporary)	Raw data	Predicted data
1971	-	-	-	2229	1592.6	-	-	-	-	-
1972	-	-	-	-	1784.4	-	-	-	-	-
1973	-	-	-	-	1996.7	-	-	-	-	-
1974	-	-	-	-	2230.7	-	-	-	-	-
1975	-	-	-	-	2487.9	47.2	1.5	-	-	-
1976	-	-	-	-	2769.5	62.7	3.6	-	-	-
1977	-	-	-	-	3076.8	97.4	8.8	-	-	-
1978	-	-	-	-	3410.6	84.8	21.3	-	-	-
1979	-	-	-	-	3771.6	11.7	50.2	-	-	-
1980	-	-	-	-	4159.9	126.4	113.5	-	-	-
1981	-	-	-	4598	4575.4	529.1	234.5	-	-	-
1982	-	-	-	-	5017.5	88.2	416.6	-	-	-
1983	-	-	-	-	5485	700.9	610.9	-	-	-
1984	-	-	-	-	5976.2	668.3	755.3	-	-	-
1985	-	-	-	-	6488.8	674.5	836.3	-	-	-
1986	-	-	-	-	7019.9	1704.1	874.7	-	-	-
1987	-	-	-	-	7566.1	1042.1	891.5	-	-	-
1988	-	-	-	-	8123.7	944.2	898.6	-	-	-
1989	-	-	-	-	8688.6	350	901.5	-	-	-
1990	-	2748.5		-	9256.3	617.6	902.7	-	-	-
1991	2412	2960.3	DWNP^a^	9032	9822.4	644.9	903.2	-	-	-
1992	-	3188.5		-	10382.5	1473.5	903.4	-	-	-
1993	1106	3434.2	DWNP	-	10932.4	1119.3	903.5	-	-	-
1994	4243	3698.9	DWNP	-	11468.2	661.1	903.5	-	-	-
1995	-	3983.9		-	11986.4	728.9	903.6	-	-	-
1996	3782	4291	DWNP	-	12484	717.3	903.6	-	-	-
1997	-	4621.6		-	12958.5	511.3	903.6	-	-	-
1998	-	4977.8		-	13408	937.1	903.6	-	-	-
1999	3886	5361.4	DWNP	-	13831.3	1359.6	903.6	-	-	-
2000	-	5774.6		-	14227.5	1248.9	903.6	-	-	-
2001	13173	6219.7	DWNP	15718	14596.2	706	903.6	-	-	-
2002	6660	6699	DWNP	-	14937.8	641.7	903.6	-	-	-
2003	5261	7215.3	DWNP	-	15252.6	368.2	903.6	-	-	-
2004	11870	7771.3	DWNP	-	15541.5	788.6	903.6	-	-	-
2005	5088	8370.3	DWNP	-	15805.6	1126.1	903.6	-	-	-
2006	9212	9015.3	DWNP	-	16046.1	622.8	903.6	-	-	-
2007	-	9710.1		-	16264.4	1021.4	903.6	-	-	-
2008	8905	10458.4	IS^b^	-	16462	1260.5	903.6	1330	405	318.6
2009	-	11264.4		-	16640.3	1345.9	903.6	1209.8	198	257.1
2010	15429	12132.6	IS	-	16800.8	1189.2	903.6	1089.7	185	207.4
2011	-	13067.6		16371	16945	755.9	903.6	969.5	-	167.4
2012	-	14074.7		-	17074.3	781.6	903.6	849.3	84	135
2013	11760	15159.4	DWNP	-	17189.9	844.5	903.6	729.2	-	109
2014	-	16327.6		-	17293.3	-	903.6	609	103	87.9
2015	-	17586		-	17385.5	-	903.6	488.8	102	70.9

^a^DWNP: Department of Wildlife and National Parks

^b^IS: Independent survey [34]

Human population census data for the study area were obtained from the Central Statistical Office (CSO), Gaborone. Since 1966, Botswana has conducted decennial population and housing censuses. We therefore considered in our analysis the number of inhabitants per village available from 1971 through to 2011.

To quantify human land use in the study area, we collected data on the number and area of agricultural fields allocated and cultivated, both from the Land Board Office and the Ministry of Agriculture, respectively. In the Okavango’s eastern Panhandle, the Land Board assigns fields exclusively for agricultural use to local farmers every year. Subsistence agriculture is the main source of livelihood in the study area, and so the majority of allocated fields are ploughed and harvested once assigned (Elizabeth Keabetswe—Head of Department of Agricultural Regional Office, Shakawe–*pers*. *comm*.). Each allocated field is measured before being given to farmers, and we used this information to calculate the total area allocated for agriculture each year. Since plots allocated are typically approximately rectangular or square, we estimated the area of each field by multiplying its width by its length. Ideally, our study should consider the area of agricultural land *cultivated* as a measure of human land use. However, this information was unavailable prior to 2008, and we therefore used the area of agricultural land *allocated* per year as a proxy. The latter, was found to be correlated with the area of land cultivated per year (Pearson’ correlation *r* = 0.87; *P* = 0.024). In other words, the area of land allocated for agricultural purposes by the Land Board represents a similar proportion of the land cultivated by farmers in the eastern Panhandle, and both (land allocated and cultivated) change equivalently in our study area. We, therefore, consider the area of agricultural land allocated (ALA, hereafter) in a year as an indicator of human land use.

Lastly, we used the number of reported raiding incidents (i.e. RRI) per year collected by the Ecoexist Project as a measure of the level of conflict occurring between people and elephants in the study area. We are aware that HEC is a complex phenomenon and that the number of crop-raiding incidents per year represents only one way to quantify the level of interaction between people and elephants. We did not consider other causes of HEC because the aim our study was to develop a preliminary baseline to assess the interaction between population sizes and land use given the overall levels of conflict in the region. Following the protocol of the International Union for the Conservation of Nature (IUCN) [36] for collection of primary data on HEC, local enumerators were selected and trained in consultation with the village Kgosi (chief) to identify and characterise elephant crop-raiding incidents in each village (for more details see [36–38]). Enumerators visited crop-raided fields following reports by farmers throughout the year. For this study, we used RRI data collected between January 2008 and April 2015.

### Statistical analysis

We performed our statistical analysis in three steps. We first investigated trends in HEC—here using the number of reported raiding incidents (RRI) as an indicator of the level of conflict between people and elephants -, and HEC drivers, i.e. human and elephant population sizes, as well as the amount of agricultural land allocated. The latter trends were estimated using data collected over two temporal scales, historical (1970s - 2015) and contemporary (2008–2015). Secondly, we assessed the relationship between each of these drivers and the level of HEC in the study area. Finally, we used the results from the previous steps to predict future levels of HEC, and we compared both temporal scales: historical versus contemporary predictions.

#### Population and HEC trends

To investigate historical trends in elephant and human population sizes, as well as in ALA area in the eastern Panhandle, we fitted statistical models that reflected the underlying structure of each raw dataset. More specifically, we applied non-linear least square logistic models (nls) to the human population growth data and to the area of agricultural land allocated (ALA). We used nls because in both cases, the relationship between human population numbers and the area of ALA as a function of years—respectively—could not be linearized by traditional statistical transformations. For the elephant population data, we used a generalised linear model (GLM) with a Poisson error structure to account for count data and a non-normal error structure. We also investigated the trend in the area of ALA during the years in which conflict data were collected (2008–2015). To do this, we used a linear model (lm) to regress the area of ALA (2008–2015) as a function of year. This model yielded normally distributed residuals over this specific period of time. In all cases, model choice reflected assumptions about the underlying data. Finally, to better understand trends in the level of conflict observed in recent years, we studied the number of reported raided incidents (RRI) per year. We used a GLM with Poisson error structure because the number of RRI corresponds to count data.

#### Temporal interaction between RRI and HEC drivers

Using the above population and HEC models we then estimated temporal trends for (1) the number of RRI per person and per elephant, (2) the area of ALA (ha) per person and per elephant, and (3) RRI per hectare of ALA each year. The objective of this analysis was to determine the intensity of conflict experienced per person and agricultural field (ha), as well as the number of raiding incidents caused by elephants inhabiting the study area. We used the same two temporal scales (historical from the 1970s - 2015; and contemporary from 2008–2015) to test the effectiveness of using long and short-term area of ALA (ha) data in assessing trends in the level of HEC.

#### Developing a model to predict future levels of HEC

We used generalised linear models with Gaussian error structures to identify which combination of the three explanatory variables considered in our analysis best described the level of conflict in the area. Human and elephant population sizes, in addition to the area of ALA (ha), were fitted as explanatory variables for the level of RRI at the historical and contemporary scales. In all cases model selection was based on corrected Akaike’s Information Criterion (AICc) [39], because of the small sample size of some of the datasets used in our study. The highest-ranked model (i.e. lowest AICc value) for each temporal scale was used to predict the area of agricultural land likely to be raided in the following ten years (from 2015 to 2025).

For all statistical analysis in this study we used R v3.0.1 [40].

## Results

### Population and HEC trends

**Fig 2:**
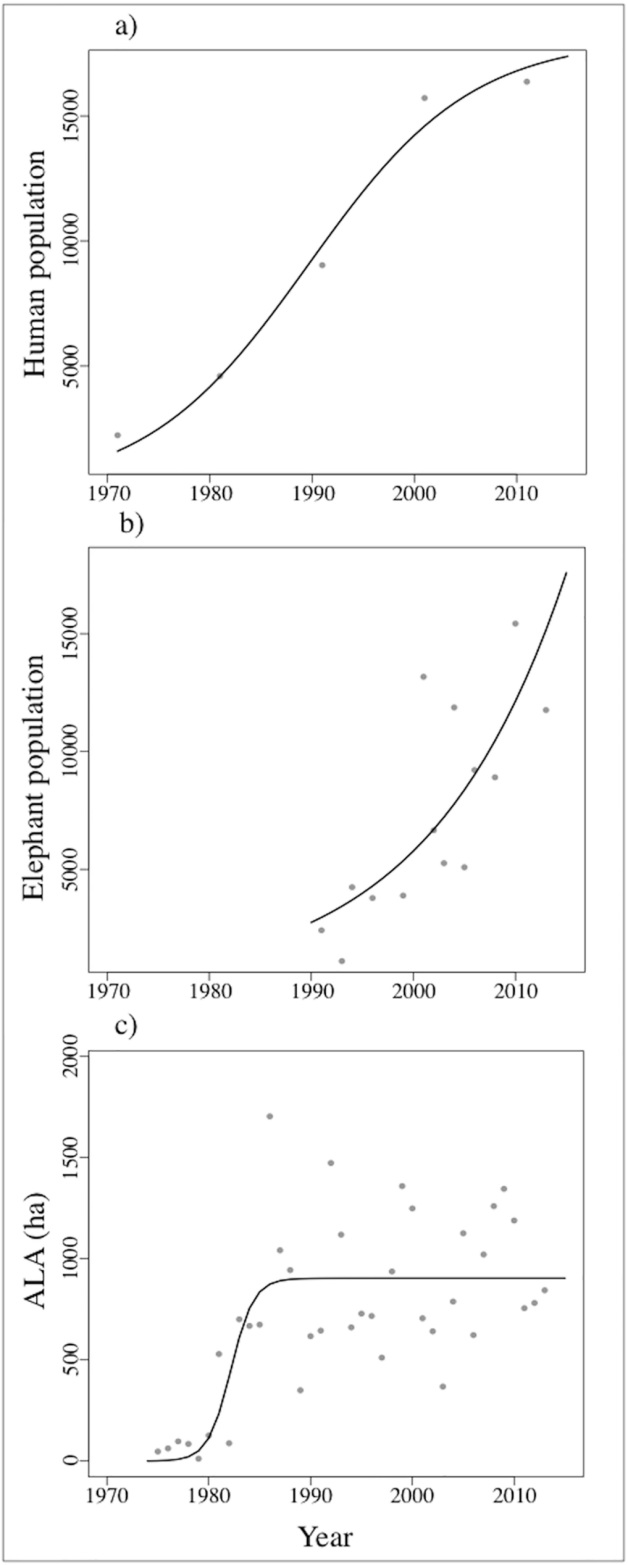
Historical trends for HEC drivers in the eastern Panhandle. **(**A) corresponds to human population, (B) elephant population, and (C) agricultural land allocated (ALA) (ha) in the Grey dots and black lines represent raw data and the best fitted model, respectively.

**Fig 3:**
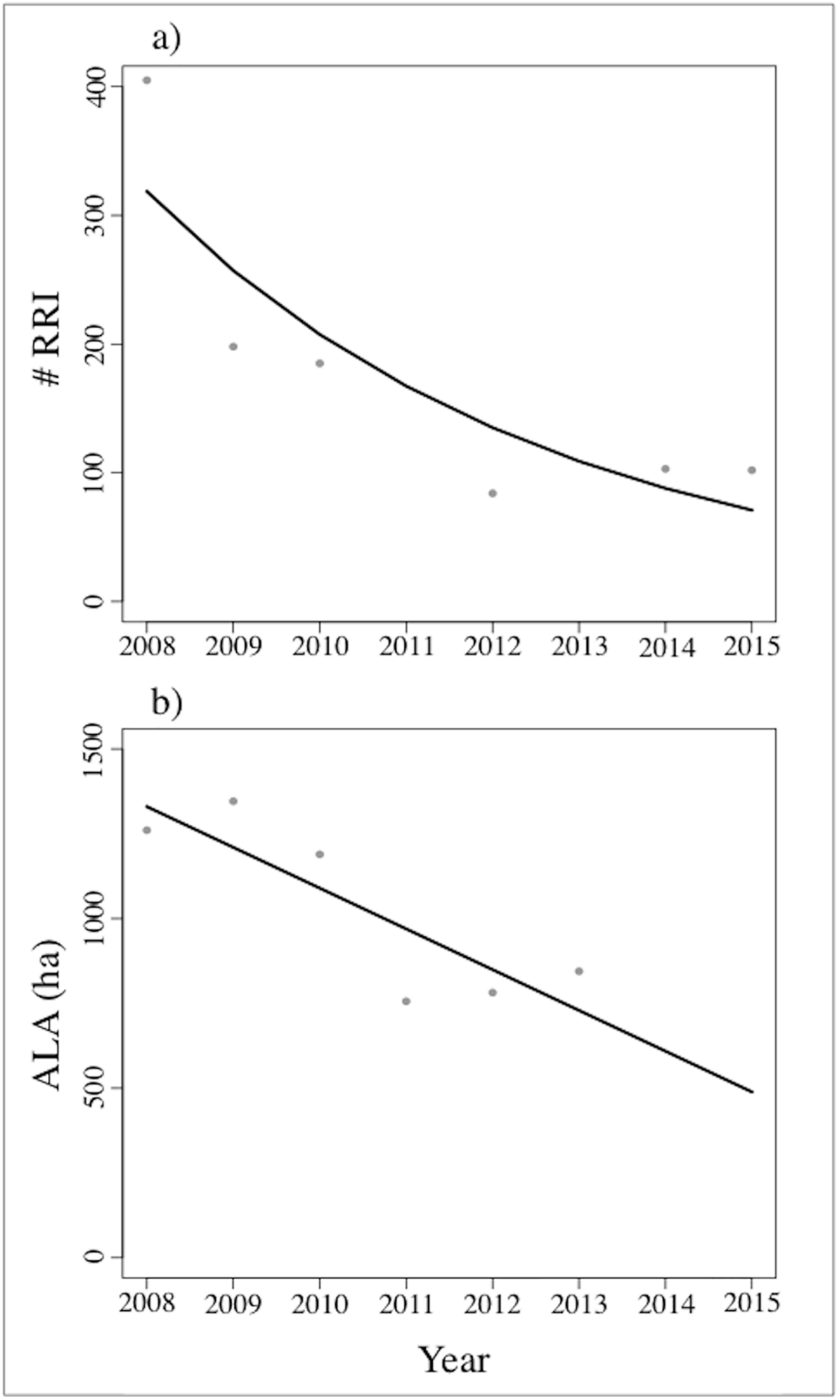
Contemporary trends for HEC drivers in the eastern Panhandle. **(**A) corresponds to agricultural allocated land (ALA) (ha), and (B) number of reported of raid incidents (RRI) in the eastern Panhandle. Grey dots and black lines represent raw data and the best fitted model, respectively.

Human and elephant populations showed increasing trends since the 1970s (Fig 2, Table 1). The number of people in the eastern Panhandle has shown a logistic growth since 1971 (Fig 2A, n = 5). This curve reached an asymptote, and in 2015 our best model predicted a local population of 17,385 inhabitants (Table 1). The elephant population, in contrast, has increased exponentially in the study area, with the population model predicting a population size of 17,586 elephants in 2015 (Fig 2B, n = 14). In contrast, the number of hectares of allocated agricultural land increased during the early 1970s in the eastern Panhandle before remaining stable from the 1980s onwards (Fig 2C, n = 40). When considering contemporary data (2008–2015), we found that the area of ALA decreased significantly (Fig 3A). Lastly, the number of yearly RRI also showed a decreasing trend between 2008 and 2015 (Fig 3B, n = 6).

### Temporal interaction between RRI and HEC drivers

**Fig 4:**
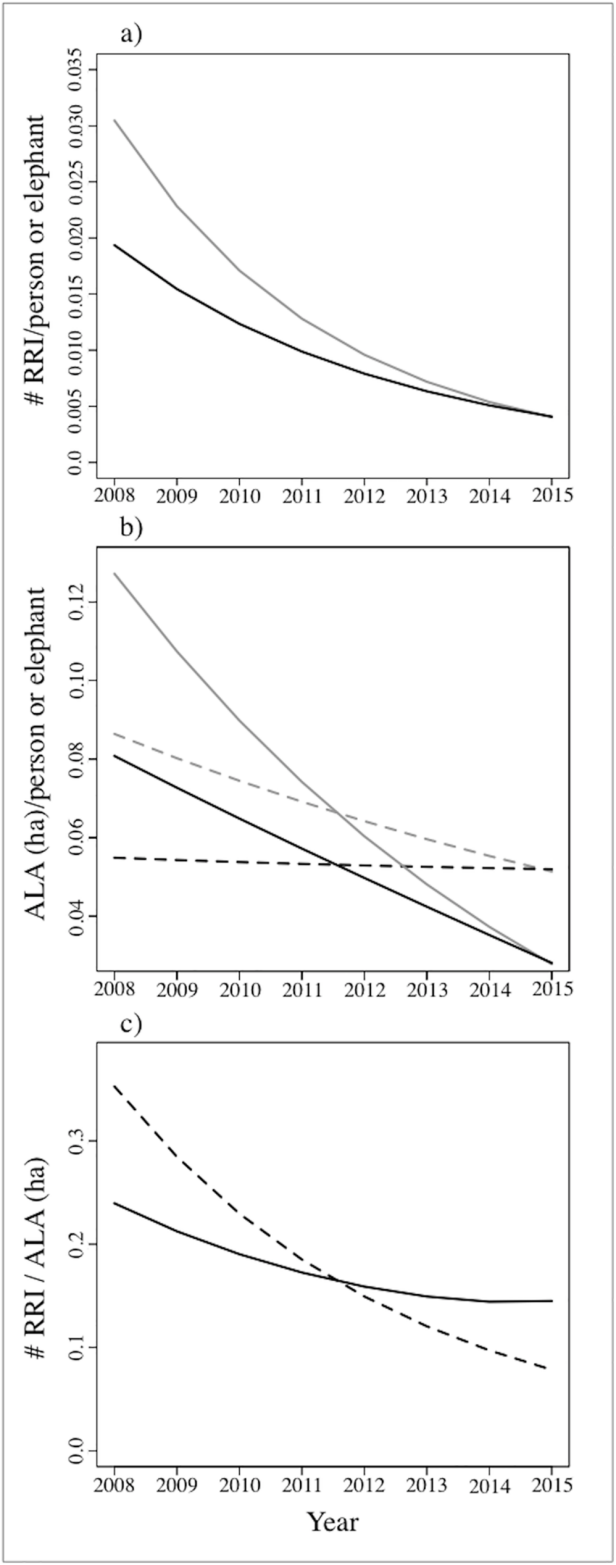
Temporal interaction between reported raid incidents and HEC drivers. (A) corresponds to the predicted number of reported raids incidents (RRI) per person (back) and per elephant (grey), (B) agricultural land allocated (ALA) per person (back) and elephant (grey); and (C) estimated raiding incidents per ha of agricultural land allocated (ALA). Dashed lines correspond to predictions from the historical model (1970s - 2015) and continuous lines to predictions from the contemporary model (2008–2015).

In accordance with the above results, the number of RRI per person and per elephant showed decreasing trends over the study period (Fig 4A). In 2008, our models predicted a number of raids per elephant of 0.03, which is in accordance with the 0.04 raids per elephant calculated from raw data. In both cases, the number of RRI decreased to 0.004 and 0.005 for fitted and raw data, respectively by 2015. A similar pattern was found for local people (Fig 4A), for which the number of RRI per person was 0.02 (from fitted values and raw data) in 2008; and in both cases respectively declined to 0.004 and 0.005 raids per person in 2015.

We detected contrasting trends for the area of ALA (ha) in the eastern Panhandle over the study period depending on which temporal scale was used. When historical data from the 1970s to the present day were used, allocated land over the study period (2008–2015) stayed constant (Fig 2C). However, when only contemporary data was considered, the trend was negative (Fig 3B). We therefore used predictions of yearly ALA from both models (historical and contemporary) to estimate: a) the hectares of ALA per person and elephant in the region, and b) the number of reported raiding incidents per ha of ALA per year. When the historical model was used to predict land-use, the number of hectares per person was found to remain constant from 0.055 in 2008 to 0.052 in 2015, but the ALA per elephant decreased from 0.087 ha in 2008 to 0.052 ha in 2015 (Fig 4B). In contrast, when the contemporary model was applied to predict land-use we observed a decreasing trend in ALA (ha) per person (from 0.08 to 0.03 ha of agricultural land (ha) per farmer) and per elephant (from 0.13 to 0.03 ha of agricultural land (ha) per elephant) (Fig 4B). Nevertheless, both models did predict a decrease in the total number of RRI per agricultural land from 2008 to 2015, albeit at different intensities (Fig 4C).

### Developing a model to predict future levels of HEC: Historical versus contemporary predictions

We used a multivariate analysis to identify explanatory variables that best explained the level of conflict in the study area at the historical and contemporary scales. Our results showed the best models to explain the amount of conflict included the additive effects of human and elephant population sizes in addition to ALA area for the historical (AICc = 47.2) the contemporary (AICc = 35.9) datasets (see Tables 2 and 3). However, because of the small sample size, we also considered models containing two explanatory variables. These models revealed that, for the historical approach, the additive effect of human and elephant population sizes were the most important predictors of conflict (AICc = 51.5), followed by the additive effect of human population and ALA (ha) (AICc = 59.3) (Table 2). In contrast, when contemporary trends where used, human population size in addition to ALA was the second best model to predict the level of conflict (AICc = 47.7), followed by the additive effects of human and elephant population sizes (AICc = 51.5) (Table 2). Independent GLMs for each explanatory variable showed human population size as the main factor affecting the trend in RRI at both temporal scales (AICc = 68.3). For both temporal scenarios, the number of people had a negative effect on the number of RRI in the region (Table 3). Similarly, RRI was negatively affected by increasing elephant population size (AICc = 84.6) at both temporal scales (Table 3). However, the area of ALA (ha) had a positive effect on the number of RRI for both, the historical (AICc = 91.7) and contemporary (AICc = 79.9) cases (Tables 2 and 3).

**Table 2:** Summary of model selection with corrected Akaike’s Information Criterion (AICc).

Temporal scale	Model	Explanatory variables	AICc	Δ AICc	Weight
**Historical** (1970s-2015)	model 1	people + elephants + ALA	47.2	0	0.893
	model 2	people + elephants	51.5	4.3	0.105
	model 3	people + ALA	59.3	12.1	0.002
	model 4	people	68.3	21.1	0.000
	model 5	elephants	84.6	37.4	0.000
	model 6	ALA	91.7	44.5	0.000
	null	1	99.4	52.2	0.000
**Contemporary** (2008–2015)	model 1	people + elephants + ALA	35.9	0	0.997
	model 3	people + ALA	47.7	11.8	0.003
	model 2	people + elephants	51.5	15.6	0.000
	model 4	people	68.3	32.4	0.000
	model 5	ALA	79.9	44.0	0.000
	model 6	elephants	84.6	48.7	0.000
	null	1	99.4	63.5	0.000

AICc used to evaluate relationships of the number of reported raid incidents with 3 explanatory variables: human (people) and elephant (elephants) population size as well as agricultural land allocated (ALA). Model selection was performed at two temporal scales, i.e. historical (1970s – 2015) and contemporary (2008–2015). The model and explanatory variables columns indicate specific combination of variables included in each function. Delta AIC (Δ AICc) shows the difference between each model and the best model selected for our analysis. The weight column represents the relative likelihood of each model.

**Table 3:** Summary of best historical and contemporary models.

Temporal scale	Best model	Coefficient	Estimate	Standard error	t value	Pr (>|t|)
**Historical** (1970s-2015)	model 1	intercept	1.90E+11	2.33E+10	8.156	0.00123
		people	-3.43E-01	8.84E-03	-38.836	2.63E-06
		elephants	-2.10E+08	2.57E+07	-8.156	0.00123
		ALA	1.24E-02	9.12E-04	13.553	0.000172
**Contemporary** (2008–2015)	model 1	intercept	1.70E+04	5.99E+02	28.36	9.19E-06
		people	-8.69E-01	2.72E-02	-31.96	5.72E-06
		elephants	-6.89E-02	5.18E-03	-13.28	0.000186
		ALA	-1.24E+00	7.34E-02	-16.93	7.13E-05

Best model coefficient estimates for the historical (1970s – 2015) and contemporary (2008–2015) analyses. The best model column corresponds to model number 1 in Table 2. Coefficient shows model intercept and explanatory variables included in the analysis: human (people) and elephant (elephants) population size, as well as agricultural land allocated (ALA). Estimate and standard error show the magnitude of each specific coefficient effect and the variation attributed to it, respectively. The t-value and Pr (>|t|) columns show the value of t-statistic and p-value for testing whether the corresponding coefficient is significantly different from 0.

**Fig 5:**
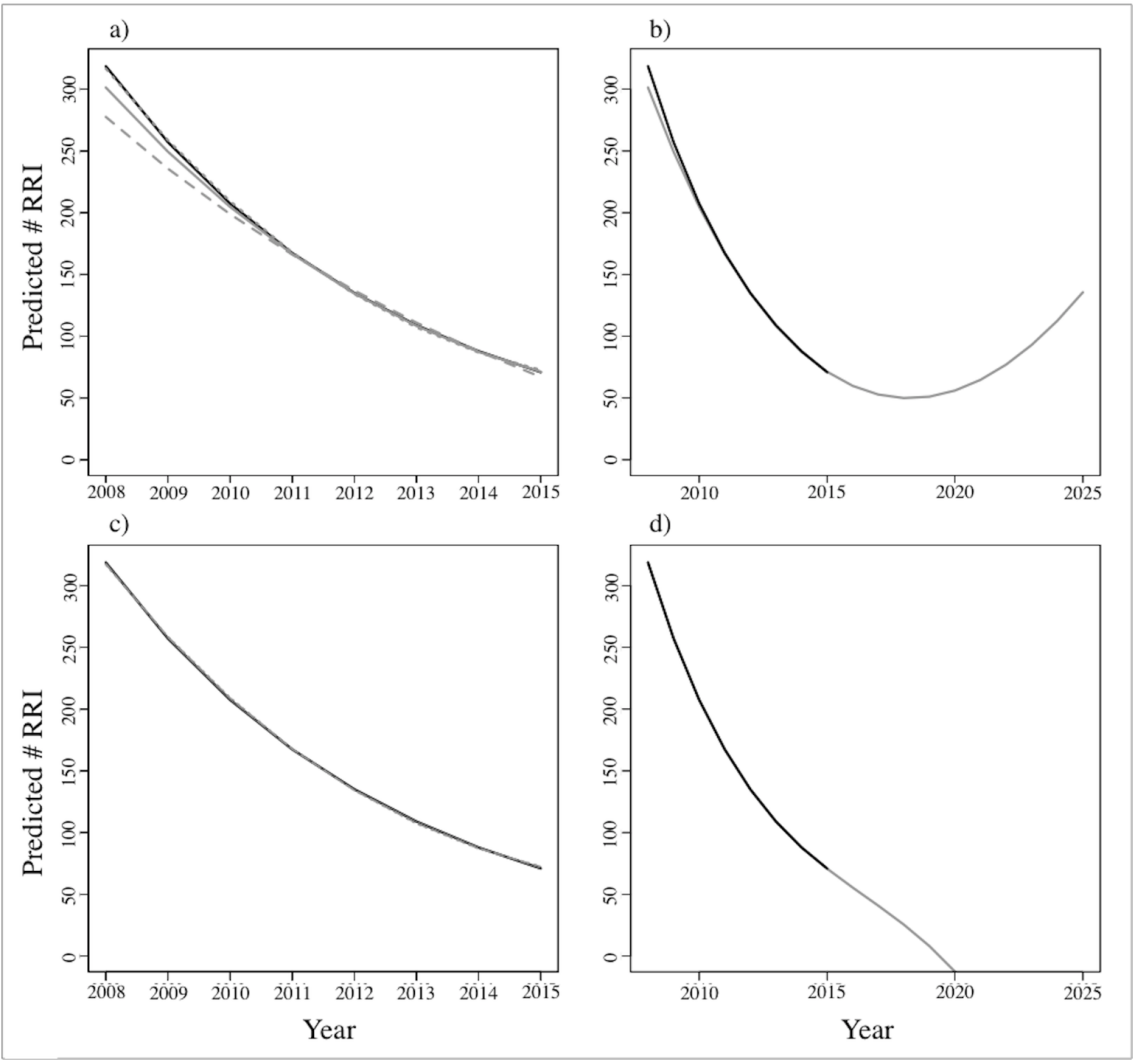
Temporal differences in predicted raiding incidents as derived from generalized linear models (GLMs). (A) and (C) show predicted reported raid incidents (RRI) from multivariate GLMs based on historical (a; 1970s – 2015) and contemporary (c; 2008–2015) data. In both graphs, grey lines represent model 1 (RRI ~ people + elephants + ALA), dotted lines model 2 (RRI ~ people + elephants), and dashed lines model 3 (RRI ~ people + ALA). (B) and (D) show RRI for the next 10 years (2015–2025) from the best GLMs (model 1) selected for historical (b) and contemporary (d) scales. Black lines correspond to raw number of RRI in all cases.

Finally, to assess the accuracy of our models we predicted the number of RRI per year with each of the best three above models (i.e. model 1–3, Table 2). In the short-term (2008–2015) the number of raids fitted did not differ significantly between both temporal scales (Fig 5A and 5C); however when the best model (from each temporal scale) was used to predict the level of conflict in the long-term we found contrasting trends (Fig 5B and 5D).

## Discussion

This study shows that inferences regarding the drivers of HEC in the eastern Panhandle and predictions for future trends are dependent on the temporal resolution of the data used. The level of reported crop raiding events by elephants in the eastern Okavango Delta Panhandle has apparently decreased in recent years, which is unexpected given that both human and elephant populations were found to be growing in the region. However, our results are in agreement with previous studies that have shown that the absolute numbers of people and elephants are not in themselves significant drivers of the conflict [5, 34]. Rather, it is the use of space and competition for resources between people and elephants that likely determines the level of conflict [2, 28, 41]. To this end, we investigated the relationship between the reported level of conflict and the area of land allocated for agriculture each year. Crucially, trends in the latter were estimated using data collected over both historical (since the 1970s) and contemporary (since 2008) timeframes, thus representing distinct data scenarios often encountered in practice [42].

When both temporal scales were considered independently our overview of the underlying processes of HEC changed, making it difficult to determine which were the most important factors contributing towards the level of conflict in the region. In line with this, the simultaneous reduction in the number of RRI and agricultural land observed since 2008 is indicative of the spatial nature of HEC [2, 28, 38], and suggests that both time and space are important in determining the likelihood of conflict [5]. The requirement for both people and elephants to share resources close to the Okavango River can be expected to result in conflict. However, our results suggest that the interaction between humans and elephants can decrease with the amount of agricultural land used by people. Thus, based on the contemporary analysis, we would concur with previous research that has considered land-use as one of the most important factors determining the level of HEC, and would re-emphasise the importance of including appropriate land-use planning in conservation management [41]. However, as previously mentioned, the contemporary model approach implemented was limited in terms of predicting conflict in the future. We therefore also investigated the interaction between HEC and the area of land allocated to agriculture based on historical data.

When this historical approach was implemented the relationship between area of land allocated to agriculture and the number of RRI became unclear. With an increasing elephant population in the study area and an unchanging allocation of land for agriculture, a decrease in the number of RRI was observed. This could be a consequence of less land allocated being cultivated, or of a switch to less palatable crops for elephant. However, we know that in the short-term the area of cultivated land has decreased at the same rate as the area of land allocated (see Supporting Information), and that, although elephants are more likely to raid fields with pumpkins, the variety and proportion of crop species planted have not drastically changed in the region, with millet being the predominant crop grown since 2008 [29]. Alternatively, changes in elephant behaviour such as moving under the cover of trees, foraging predominantly during the night, and the use of pathways [29, 41, 43–46], could help explain the decrease in HEC when trends in land-use are estimated from historical data. However, although we acknowledge the importance of behavioural components–which we did not include in our study–we argue it is more likely that the trend in RRI observed within the last decade is a reflection of population and land-use factors acting in the short term, which may be different to those acting over longer time-scales.

It is interesting to highlight that the use of different time scales when determining the area of land allocated for agriculture did not impact the estimation of temporal trends in conflict in the short-term. In contrast, disparities were found when predicting in the long-term. In both cases, the number of RRI per person, elephant and agricultural land (ha) decreased between 2008 and 2015. Accordingly, our multivariate analysis predicted minor dissimilarities between temporal scales, both of which resulted in an estimated decrease in the level of conflict. However, when the same models were applied to predict HEC in future years (2015–2025) we found opposite patterns between the two temporal scales. Such a result is likely due to changing trends of land-use at different time scales, and therefore we think these contrasting outcomes are a good example of the ‘shifting baseline syndrome’. This concept was first introduced by Pauly (1995), who described how each generation of scientists set a baseline that started at the beginning of their careers, and used it to evaluate changes in the future. However, so-called shifting baselines masked longer-term changes. In our case, analyses based on land-use estimates derived from 2008 predicted an end to the conflict by 2020. However, when long-term data were considered, HEC was predicted to increase in the future.

The question of which temporal scale is preferable to use when predicting HEC is a challenging one to answer because natural systems are often extremely dynamic and their component interactions complex. Instead, we argue that both should be used as complementary sources of information with which to better investigate the underlying processes driving HEC. Ideally, baselines should be determined before decision-making takes place, thus our study represents an extremely useful framework to face future challenges in elephant conservation. In addition to time scale, we encourage detailed analyses of human behaviour (i.e. peoples’ livelihood, migration history, etc.), elephant behaviour (habitat preferences, movement patterns, etc.) and land-use (effective cultivated land, crops yielded, etc.) to be carried out so as to provide a more comprehensive understanding of additional drivers of HEC in the study area.

Nonetheless, HEC is a complex phenomenon driven not only by population and land use trends, but also historical, social, political, cultural and environmental factors that are unique to each study area. The aim of our study was not to include all of these, and we acknowledge that aspects other than population trends may be contributing towards the observed trend in conflict. For instance, a common problem in HEC is the lack of reporting of crop-raiding incidents, which could represent a potential source of bias in our measurement of HEC. Compensation schemes, which reimburse individuals who experience property damage or have been injured by wildlife in the hope of increasing tolerance towards wildlife [47], may inadvertently increase the likelihood of reporting. Alternatively, and most likely in the case in our study area, farmers may feel that the compensation is inadequate [28–29, 48], and we suspect local people could be reporting less than they used to in the eastern Panhandle. The implementation of compensation schemes is challenging and in many cases difficult to monitor [47, 49–52], and the compensation programme in Botswana is not an exception in terms of difficulties in the field [47, 49–50]. Nevertheless our measure of conflict is liable to misreporting, it is often the only source of information in affected areas, and therefore we used it as the only conservative long-term indicator of the conflict status. Importantly, however, our study considers a single measure of HEC (i.e. crop-raiding reports), and thus, future research should ideally consider other sources of biases associated with the measurement of HEC, which may also potentially influence observed trends.

Northern Botswana holds the largest population of elephants in the world, therefore a decline in reported HEC in the Okavango Delta Panhandle can be seen as very encouraging, particularly given human and elephant populations are still increasing, and agricultural land in the long-term is still expanding. Our study revealed that the characterisation of HEC drivers was dependent on the time scale used to estimate allocated land-use over the past eight years. Specifically, it was likely to change considering the fluctuating amount of land allocated to agriculture over the past three decades, thus highlighting the spatial-temporal nature of HEC in the study area and leading us to the tentative conclusion that the decreasing trend in RRI is probably linked to the reduction of ALA in the eastern Panhandle since 2008. More generally, it is essential for conservationists to consider baselines at different temporal scales before making decisions about management and species conservation. In our study area, if larger areas were used for agriculture in the future, the trend in HEC would also change. We therefore emphasize it is critical to design effective land-use planning programs in the short-term in order to minimise and prevent future conflict between people and elephants in the future.

## Supporting information

S1 Fig Area of land cultivated as a proportion of agricultural land allocated in the eastern Panhandle between the years 2008–2014. (TIFF)
