# Supplementary figures and images for: Determining baselines for human-elephant conflict: A matter of time
Source: PLoS One. 2017 Jun 5;12(6):e0178840. doi: 10.1371/journal.pone.0178840 (PMC5459443; doi:10.1371/journal.pone.0178840)

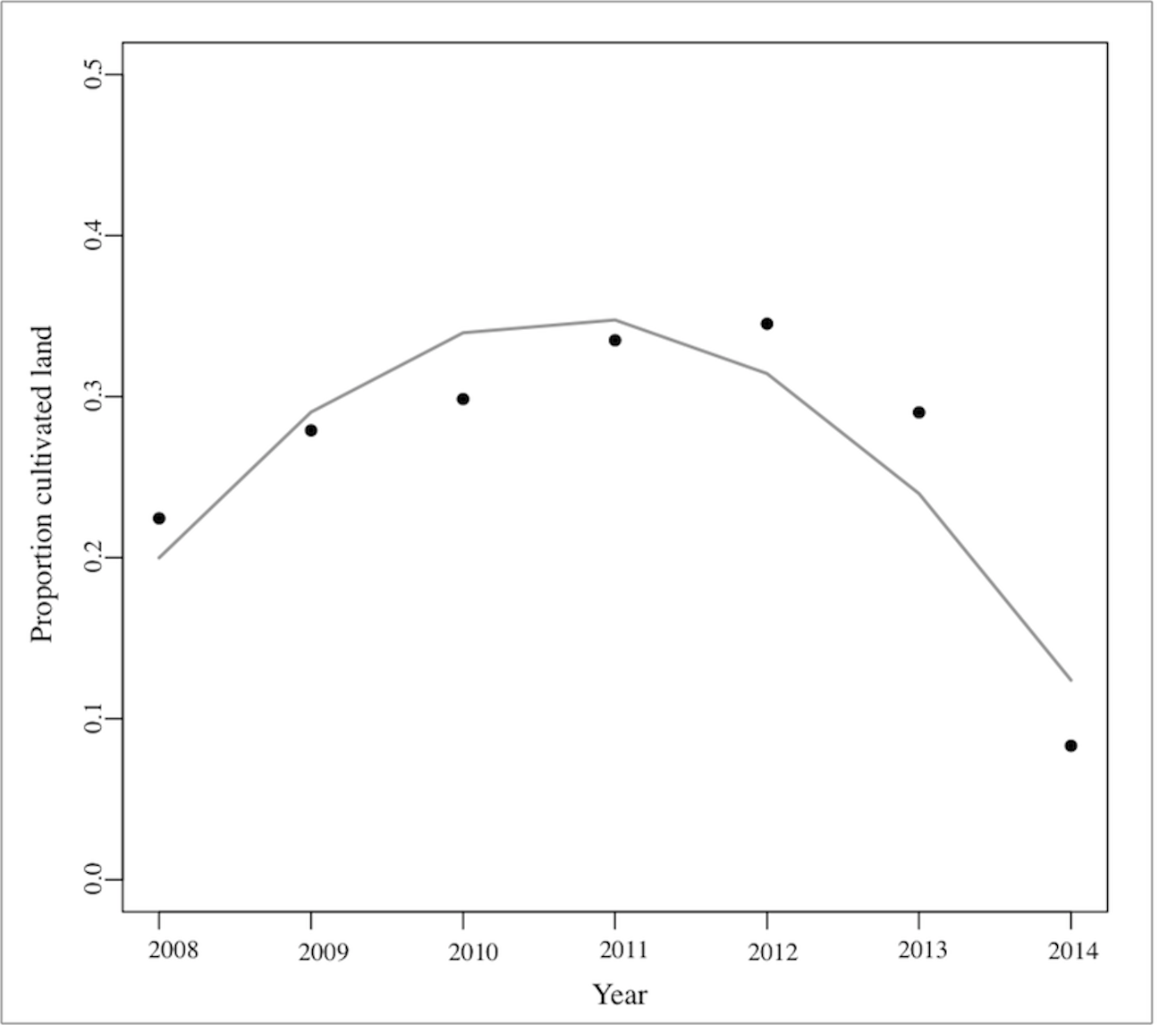

Supplement: S1 Fig — (TIFF) [file pone.0178840.s001.tiff]
